# The Structural and Molecular Mechanisms of *Mycobacterium tuberculosis* Translational Elongation Factor Proteins

**DOI:** 10.3390/molecules29092058

**Published:** 2024-04-29

**Authors:** Ning Fang, Lingyun Wu, Shuyan Duan, Jixi Li

**Affiliations:** 1 State Key Laboratory of Genetic Engineering, School of Life Sciences, Shanghai Engineering Research Center of Industrial Microorganisms, Fudan University, Shanghai 200438, China; 21210700108@m.fudan.edu.cn (N.F.); 22307110421@m.fudan.edu.cn (L.W.); 2 College of Food Science and Pharmaceutical Engineering, Zaozhuang University, Zaozhuang 277160, China

**Keywords:** *Mycobacterium tuberculosis*, translation factor proteins, EF-Tu, EF-Ts, EF-G, structural, molecular mechanism

## Abstract

Targeting translation factor proteins holds promise for developing innovative anti-tuberculosis drugs. During protein translation, many factors cause ribosomes to stall at messenger RNA (mRNA). To maintain protein homeostasis, bacteria have evolved various ribosome rescue mechanisms, including the predominant trans-translation process, to release stalled ribosomes and remove aberrant mRNAs. The rescue systems require the participation of translation elongation factor proteins (EFs) and are essential for bacterial physiology and reproduction. However, they disappear during eukaryotic evolution, which makes the essential proteins and translation elongation factors promising antimicrobial drug targets. Here, we review the structural and molecular mechanisms of the translation elongation factors EF-Tu, EF-Ts, and EF-G, which play essential roles in the normal translation and ribosome rescue mechanisms of *Mycobacterium tuberculosis* (Mtb). We also briefly describe the structure-based, computer-assisted study of anti-tuberculosis drugs.

## 1. Introduction

*Mycobacterium tuberculosis* (*M. tuberculosis*, Mtb), the causative agent of tuberculosis (TB), was discovered by Robert Koch in 1882 [1]. Despite the introduction of various anti-tuberculosis drugs over the years, TB remains one of the leading causes of death worldwide, and TB treatment requires a combination of multiple antibiotics, which is a time-consuming process that leads to the development of drug-resistant TB [2]. Mtb possesses a unique mechanism for establishing a latent tuberculosis infection, called non-replicative dormancy, which can persist in the host even in the presence of a functional immune response. The disease-causing mechanism of Mtb depends on its ability to successfully block the innate defenses of host macrophages [3]. The emergence and spread of multidrug-resistant strains has escalated antibiotic resistance to a critical public health concern [4]. It is estimated that one quarter of the world’s population is latently infected with Mtb, and 5–10% of those infected develop acute infection [5]. However, existing drugs have not proven effective in eradicating Mtb from latent lesions. Furthermore, the current vaccine-*Mycobacterium* bovis Bacillus Calmette–Guérin (BCG) only prevents severe childhood (age < 15 years old) TB, offering no protection against adult TB [3]. Therefore, it is urgent to find new anti-tuberculosis drugs.

**Figure 1:**
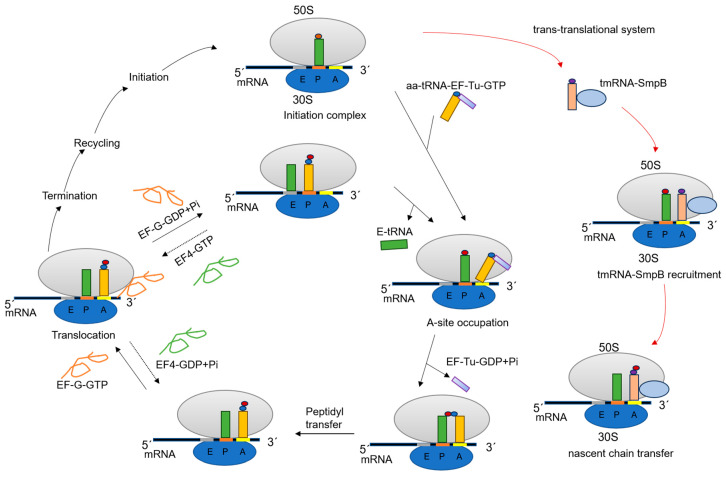
Model diagram of the functional stages of the ribosome during translation. The 70S complex contains the initiating tRNA at the P-site of the ribosome. The 70S complex binds to the aa-tRNA-EF-Tu-GTP ternary complex and enters the elongation cycle. Subsequently, GTP is hydrolyzed, EF-Tu-GDP and inorganic phosphate (Pi) leave the ribosome, and aa-tRNA enters the A-site. The nascent peptide chain is transferred from the P-site to the A-site, resulting in a one amino acid extension of the peptide chain. In the presence of EF-G-GTP, the ribosome moves one codon away from the 3’ end of the mRNA, and the diacyl peptide-tRNA in the A-site moves to the P-site. EF4-GTP catalyzes the reversal of this step. When the stop codon enters the A-site, protein synthesis is terminated with the assistance of release factors. Also, some of the key steps in the rescue mechanism are labeled with red arrows in the figure.

Protein synthesis, or gene translation, is a key step at the heart of molecular biology and is implemented by the ribosomes of all cells, which decode the genetic information in the mRNA into polypeptides through this process. Remarkably, nearly 40% of known antibiotics target the ribosome itself [3,6]. During latent TB infection, the persistence of non-replicating Mtb slows all cell processes, including translation, diminishing the effectiveness of antibiotics that target the ribosome in treating TB [7]. The protein translation process in prokaryotes consists of four main steps: initiation, elongation, termination, and recycling (Figure 1). After termination, mRNA and transfer RNA (tRNA) are released from the ribosome to separate the 30S and 50S subunits and initiate a new translation round. Most ribosomes complete the translation process accurately and completely [8] by delivering aminoacyl-tRNA (aa-tRNA) to ribosomes programmed with mRNA using the thermo-unstable Tu factor (EF-Tu), then using elongation factor G (EF-G) to help the tRNA-mRNA complex translocate on the ribosome (Figure 1) [9,10]. When ribosomes encounter obstacles to translation, such as chemical damage to mRNA caused by the environment or cellular agents, they stall on the mRNA rather than continuing to synthesize proteins [11,12,13,14]. These obstacles form so-called ‘no-go complexes’ that prevent ribosomes from proceeding on the mRNA, which is very harmful to the cell because it not only affects protein translation but is also likely to produce harmful proteins [11]. Even under normal growth conditions, about 2–4% of translation is targeted by the ribosome rescue system [15].

In this regard, bacteria have specialized rescue systems. The predominant and distinctive system is the trans-translational system mediated by transfer messenger RNA (tmRNA, 10SaRNA, SsrA) and small protein B (SmpB) (Figure 1) [16]. Additionally, some bacteria have other rescue systems that do not depend on tmRNA to function, such as ArfA/RF2 [17] and ArfB (YaeJ) [18]. tmRNA is a special RNA with both tRNA and mRNA properties. The initial confirmation of sequence similarity between the tmRNA and tRNA parts was based on the tmRNA gene sequence of Mtb [19]. In the trans-translation rescue system, after binding to SmpB, the aminoacylated tmRNA binds to the hollow A-site of the stalled ribosome with the help of the elongation factor EF-Tu. The ribosome resumes translation, adds a degradation tag to the nascent peptide, and ultimately recycles the stalled ribosome and degrades the non-terminating mRNA and the aberrant proteins it encodes [20]. Trans-translation is essential for the survival of numerous pathogens, including Mtb, *Helicobacter pylori*, *Neisseria gonorrhoeae*, and *Shigella flexneri* [21,22,23,24], and is ubiquitous in bacteria, but absent in animals or humans [25,26,27]. In Mtb, this is the only mechanism of ribosome rescue that has been identified. Therefore, targeting this pathway could present a promising strategy to address diverse, severe, and pressing microbial threats [4].

The overall ribosomal structure of Mtb and its close relative *Mycobacterium smegmatis* (*M. smegmatis*) is similar to that of other bacteria. *M. smegmatis* is a non-pathogen and is an ideal model system for the study of Mtb [3]. Both the protein synthesis process and the ribosome rescue pathway require translation elongation factors (EFs), the essential catalysts, to assist the ribosome in achieving accurate and efficient protein synthesis [4]. All stages are mediated by specific factors, some of which are bacteria-specific, while others (e.g., elongation factors EF-Tu and EF-G) are universally conserved across species. Prokaryotic systems employ three primary translation EFs: EF-Tu, EF-Ts, and EF-G. These factors are frequent targets for antibacterial drugs due to their significance in cellular processes. In recent years, with the rapid development of cryo-electron microscopy, high-resolution structures of translation elongation factors and ribosome complexes have been obtained, which, together with the high-resolution structures of translation elongation factor monomers and complexes obtained by X-ray diffraction in recent years, have provided favorable support for an in-depth understanding of the biological functions of translation factors and thus, the development of new antimicrobial drugs. In this review, we will give an overview of the structure and mechanism of Mtb translation elongation factors EF-Tu, EF-Ts, and EF-G. We will also briefly summarize the development of structure-based, computer-assisted anti-tuberculosis drugs.

## 2. The Role of EF-Tu and EF-Ts in Mtb

EF-Tu is an important and universally conserved GTPase and an essential translational element that promotes protein biosynthesis in the ribosome [4,9,28,29]. During protein biosynthesis, EF-Tu binds to GTP and directs aa-tRNA to the vacant A-site of the ribosome in the form of EF-Tu-GTP-aa-tRNA ternary complex (Figure 1) [30,31]. EF-Tu undergoes a conformational change when the codon of the mRNA undergoes codon–anticodon pairing with the anticodon of the aa-tRNA, which then hydrolyzes GTP to GDP and inorganic phosphate (Pi) [32,33].

Following GTP hydrolysis, EF-Tu-GDP is released from the ribosome as an inactive form, allowing aa-tRNA to enter the A-site for protein synthesis [34]. Next, elongation factor thermo-stable factor (EF-Ts) binds to EF-Tu, facilitating the exchange of GDP with GTP, regenerating the EF-Tu-GTP complex, delivering another aa-tRNA, and then starting a new translation cycle (Figure 1) [35]. Despite the equalizing effect of EF-Tu, translation remains a discontinuous process with translation stalling. One important reason for this is the presence of proline residues on the nascent peptide chain. Proline forms proteins more slowly than other amino acids, and tripeptides containing two consecutive prolines are the shortest and most common sequence leading to ribosomal stalling [36]. To address this, the bacterial translation elongation factor P (EF-P) relieves this stalling and allows protein biosynthesis to continue [37].

Across all species, ribosomes synthesize proteins by faithfully decoding mRNA nucleotide sequences with an aminoacyl-tRNA substrate. The current understanding of the decoding mechanism comes mainly from studies of bacterial systems. In humans, changes in decoding fidelity are associated with aging and disease, presenting potential points of therapeutic intervention in viral and cancer therapy. While the decoding process in humans closely resembles that in bacteria, the response dynamics for aminoacyl-tRNA movement within human ribosomes differ significantly. This alteration operates at a pace approximately an order of magnitude slower than in bacteria, resulting in a more precise mRNA decoding process in humans [38]. These differences arise from eukaryotic-specific structural elements and eukaryotic elongation factor 1A (eEF1A) in human ribosomes, which coordinate faithful tRNA doping at each mRNA codon [38]. The counterpart protein of eEF1A in bacteria is EF-Tu, thus showing that the translational elongation factor EF-Tu (or eEF1A) is essential both in eukaryotes and in prokaryotes.

In addition, EF-Tu plays an important proofreading role in promoting efficient protein synthesis [39]. In the trans-translational system, the tmRNA encoded by the ssrA gene interacts with SmpB, EF-Tu, and the ribosomal protein S1 (RpsA), to efficiently release stalled ribosomes through the formation of ribonucleoprotein complexes [40]. During trans-translation, EF-Tu transports alanyl-tmRNA-SmpB to the empty A-site of the ribosome [4]. Like a typical tRNA transport, the elongation factor Tu (EF-Tu-GTP) carries the alanyl-tmRNA-SmpB complex into the stagnant ribosome during trans-translation [16]. In Mtb, EF-Tu is a key component of the initial trans-translation step, and trans-translation is considered a valid and promising target for the development of new antibiotic drugs to shorten the duration of TB treatment, such as the first-line TB drug pyrazinamide that inhibits trans-translocation of Mtb [41,42,43]. Given the critical role of EF-Tu in Mtb under normal growth conditions and stress conditions, and the fact that various types of antibiotics targeting EF-Tu can alter the viability of Mtb by blocking the function of EF-Tu, Mtb EF-Tu is generally considered a highly promising molecular target for rational drug design in tuberculosis [42].

### 2.1. Structure Alignment of Mtb EF-Tu and EF-Ts with Those of Other Bacterial Sources

The structures of EF-Tu proteins originated from various bacteria have been reported, including EF-Tu from *Thermus thermophilus* [44], *Escherichia coli* [45], *Thermus aquaticus* [46], *Pseudomonas putida* [47], *Pseudomonas aeruginosa* [48], *M. tuberculosis* [49], *Saccharolobus solfataricus* [50], and *Thermotoga neapolitana* [51]. In almost all structures, EF-Tu forms a complex with other components such as GTP, GDP, EF-Ts, tRNAs, and ribosomes involved in protein synthesis (Table 1). Among them, there are abundant reports on the structure of EF-Tu in *E. coli* and *T. thermophilus* (Table 1).

**Figure 2:**
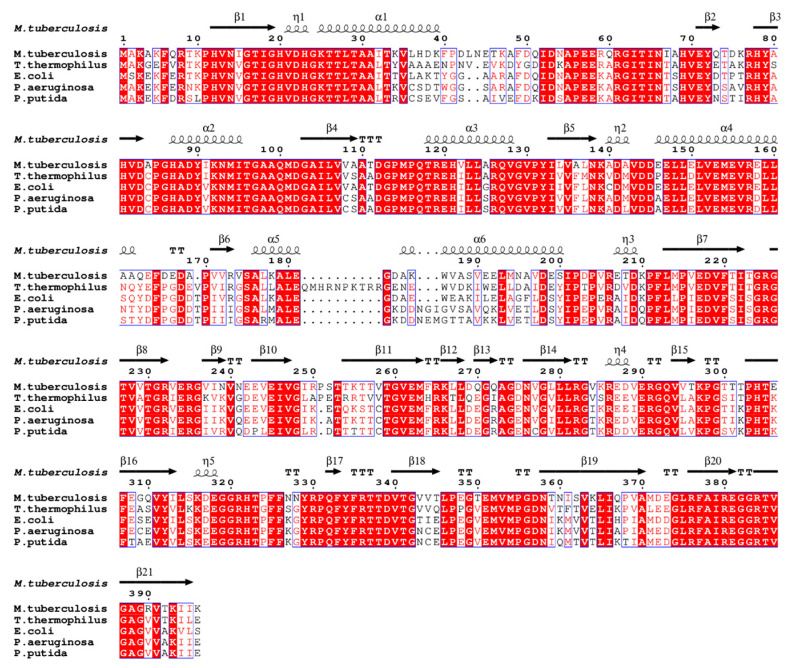
EF-Tu sequence alignment among different species using ENDscript server [53]. Identical and similar residues among groups are shown in white font on a red background and in red font on a white background, respectively.

**Figure 3:**
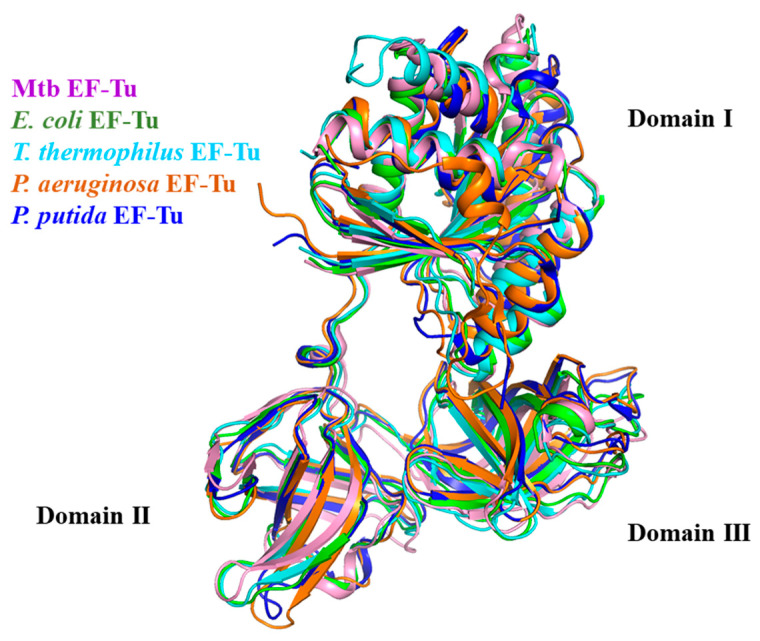
EF-Tu structure alignment among Mtb (PDB:7VOK, light pink), *E. coli* (PDB: 1EFU, green), *T. thermophilus* (PDB: 1AIP, cyan), *P. aeruginosa* (PDB: 4ZV4, orange), and *P. putida* (PDB: 4J0Q, blue).

The primary sequence of EF-Tu is highly conserved during evolution (Figure 2) [2]. The alignment of the EF-Tu structure among Mtb, *E. coli* (RMSD: 2.155 Å), *T. thermophilus* (RMSD: 2.550 Å), *P. aeruginosa* (RMSD: 1.249 Å), and *P. putida* (RMSD: 1.016 Å) has a similar architecture (Figure 3) [49]. These structures show that EF-Tu contains three domains. Domain I, of ~200 residues at the N-terminus, contains the GTPase center, which is responsible for binding to GTP or GTP analogs. The GDP molecule interaction residues were conserved among Mtb, *E. coli*, *T. thermophilus, P. aeruginosa*, and *P. putida* [49]. Domain II, of ~100 residues following domain I, works together with domain III, of ~100 residues in the C-terminal of EF-Tu, to bind aa-tRNA with EF-Ts [35]. Structural domains I and III are closer together than structural domains I and II, allowing domains I and III to meet face-to-face, leading to side-chain interactions (Figure 3) [49]. In addition, the flat triangle formed by the three domains ensures a high degree of interdomain flexibility, which facilitates the binding of EF-Tu to various substrates during peptide synthesis [52].

**Figure 4:**
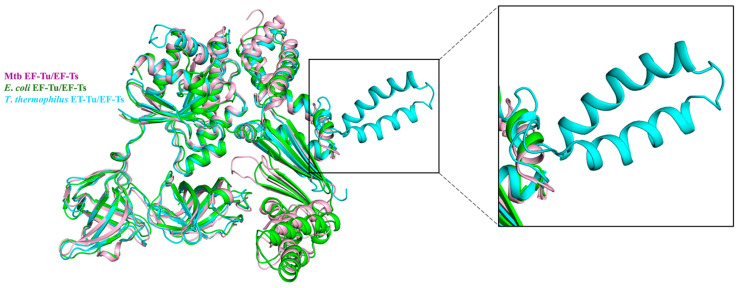
EF-Tu/EF-Ts complex structure alignment among Mtb (PDB:7VMX, light pink), *E. coli* (PDB: 1EFU, green), and *T. thermophilus* (PDB: 1AIP, cyan). The zoomed-in area shows the detailed difference of EF-Ts among the three species. The *T. thermophilus* EF-Ts had one extra helix compared to the other two species.

In contrast to the high conservation of EF-Tu, EF-Ts were less similar among different species [49]. The proportion of EF-Ts that bound and reactivated with EF-Tu differed between Mtb and *T. thermophilus* [44,54]. The alignment of the complex structure EF-Tu/EF-Ts among Mtb, *E. coli*, and *T. thermophilus* shows that EF-Ts bind mainly to domain I of EF-Tu in all three structures (Figure 4). The structure comparison between Mtb EF-Tu/EF-Ts and *E. coli* EF-Tu/EF-Ts is highly similar, with an RMSD value of 1.721 Å (Figure 4). However, the main difference is that in *E. coli*, the C-terminal domain of EF-Ts is stretched to interact with EF-Tu domain I, which is absent in EF-Ts from Mtb (Figure 3 and Figure 4) [44,55]. Unlike Mtb EF-Ts and *E. coli* EF-Ts, the *T. thermophilus* EF-Ts had one extra helix (Figure 4) [49]. The structure of Mtb EF-Tu in the complex EF-Tu•GDP and EF-Tu•EF-Ts is nearly identical, with an RMSD value of 0.711 Å. A significant difference is that EF-Tu in the EF-Tu•EF-Ts complex had a much more compact shape [49].

**Figure 5:**
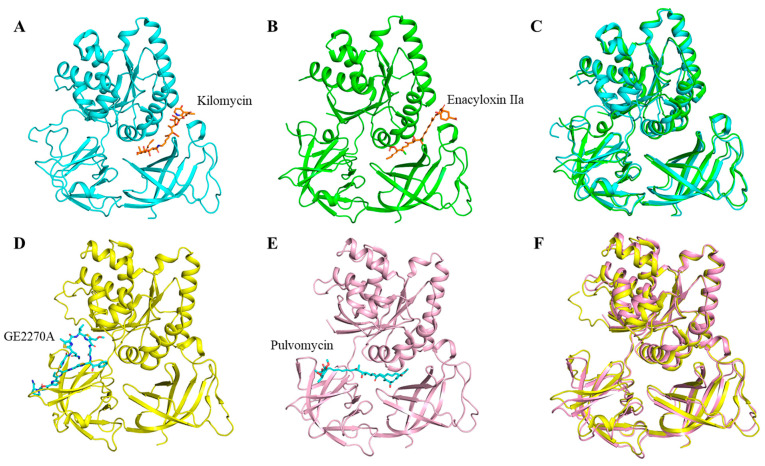
The complex structures of EF-Tu (shown in cartoon) and its according antibiotics (shown in stick models). (**A**) Kilomycin binds to *E. coli* EF-Tu (PDB:1OB2, cyan). (**B**) Enacyloxin IIa binds to *E. coli* EF-Tu (PDB:2BVN, green). (**C**) Structure alignment between (**A**) and (**B**). (**D**) GE2270A binds to *T. thermophilus* EF-Tu (PDB:2C77, yellow). (**E**) Pulvomycin binds to *T. thermophilus* EF-Tu (PDB:2C78, light pink). (**F**) Structure alignment between (**D**) and (**E**).

The structures of the complexes of several antibiotics with EF-Tu have been reported in the protein data bank (PDB) database, among which more studies have been carried out on four classes of antibiotics, namely kirromycin, enacyloxin IIa, pulvomycin, and GE2270A (Table 2). Kilomycin and enacyloxin IIa inhibit the release of EF-Tu-GDP from the ribosome, and their binding sites to EF-Tu are located at the junction of structural domain I and domain III of EF-Tu (PDBs:2BVN, 1OB2) (Figure 3 and Figure 5) [56,57]. Compared to kirromycin (PDB:1OB2), enacyloxin IIa (PDB:2BVN) makes the structure of EF-Tu more compact between domain I and domain II (Figure 5A,C), with an RMSD value of 0.954 Å. Pulvomycin and GE2270A prevented the binding of EF-Tu•GTP to aa-tRNA [57,58,59], and their binding sites to EF-Tu are mainly located at the domain II of EF-Tu (PDBs:2C77, 2C78, 1D8T) (Figure 3 and Figure 5). Compared to pulvomycin (PDB:2C78), GE2270A (PDB:2C77) makes the structure of EF-Tu more compact between domain I and domain II (Figure 5D–F), with an RMSD value of 0.954 Å. In addition, new mechanisms of antibiotic action have been reported in recent years, such as the antibiotic KKL-5 which inhibits translation by blocking the binding of EF-Tu to tmRNA, indicating that the inhibition of EF-Tu-tmRNA binding is a viable option for antibiotic development [4]. However, the structure of the Mtb EF-Tu in complex with antibiotics has not been reported yet, so obtaining the structure of this complex in the future will provide an important theoretical basis for the development of new anti-tuberculosis drugs.

### 2.2. The Role of EF-G in Mycobacterium tuberculosis

During protein translation, a conserved GTPase EF-G in bacteria, or its counterpart elongation factor 2 (eEF2) in eukaryotes, transports tRNA and mRNA through the ribosome [46]. Blocking GTP hydrolysis after the movement of mRNA and tRNA essentially and completely cancels the rotation of the structural head domains of the 30S ribosome. Meanwhile, GTP hydrolysis critically prevents the release of EF-G before the tRNA and mRNA have been moved by a complete codon and ensures productive translation and the maintenance of translated reading frames [60]. To elucidate how the nearly rigid EF-G corrects the inherent spontaneous dynamics of the ribosome in tRNA-mRNA translation, and how GTP hydrolysis and Pi release drive the dissociation of EF-G, time-resolved cryo-electron microscopy was used to visualize GTP-catalyzed translocation in the absence of an inhibitor [46]. The high-resolution ribosome-EF-G intermediate structure showed that, before translocation, EF-G binds to peptidyl-tRNA and the rotating ribosomal 30S subunit stabilizes the GTPase catalytic center of EF-G [46]. The reverse rotation of the 30S subunit releases Pi and translocates peptidyl-tRNA and EF-G by about 20 Å. An additional 4 Å translocation allows EF-G to be dissociated from the transient, highly rotated, head domain of the 30S subunit. The additional 4 Å translocation allows EF-G to dissociate from the highly rotated transient ribosomal state of the head domain of the 30S subunit [46].

EF-G is the only classical GTPase that can act at two different stages of protein synthesis, showing a common GTPase activation mechanism and ribosome binding mode [61]. As an essential protein in protein synthesis, EF-G facilitates ribosome translocation during the elongation phase and promotes the movement of ribosomes along the mRNA template for accurate and efficient translation of the genetic code into protein (Figure 1) [62]. During peptide translocation, EF-G performs by shifting the polypeptide chain from the A-site to the P-site; and, during protein synthesis, EF-G interacts with the ribosome release factor (RRF) to release the ribosome complex for recycling [63,64,65].

In addition, during trans-translation, the SmpB protein forms a complex with tmRNA, and the transporter-like ribonucleic acid structural domain (TLD) of tmRNA enters the A-site of the ribosome. Subsequently, driven by EF-G, the TLD-SmpB module is translocated to the P-site [66]. Meanwhile, trans-translation stalled ribosomes can be rescued by a ubiquitously conserved GTPase in bacteria called EF4, which has about 70% sequence similarity to EF-G. This factor recognizes stalled ribosomes with deacetylated tRNAs at the E-site and peptidyl-tRNAs at the P-site and catalyzes the inversion reaction [10]. GTP cleavage is not important for tRNA movement, but EF-G-mediated translocation in the presence of GTP is at least four times faster than the non-hydrolyzable GTP analog GDPNP [10]. How this acceleration is achieved remains unclear. Hydrolysis of GTP is believed to mainly be important for the rapid and efficient release of EF-G, which is essential for binding the incoming ternary complex to the ribosome [10]. Compared to the ribosome in the classical state ribosome [67], the 70S-tmRNA-EF-G complex has undergone large-scale conformational changes [66]. In Mtb, EF-G is a promising target for drug design [68]. Fusidic acid (FA), a drug widely used to treat tuberculosis in the clinic, inhibits Mtb protein synthesis by binding to EF-G and preventing its release after GTP hydrolysis and translocation [68,69].

### 2.3. Structure Alignment of Mtb EF-G with Those of Other Bacterial Sources

**Figure 6:**
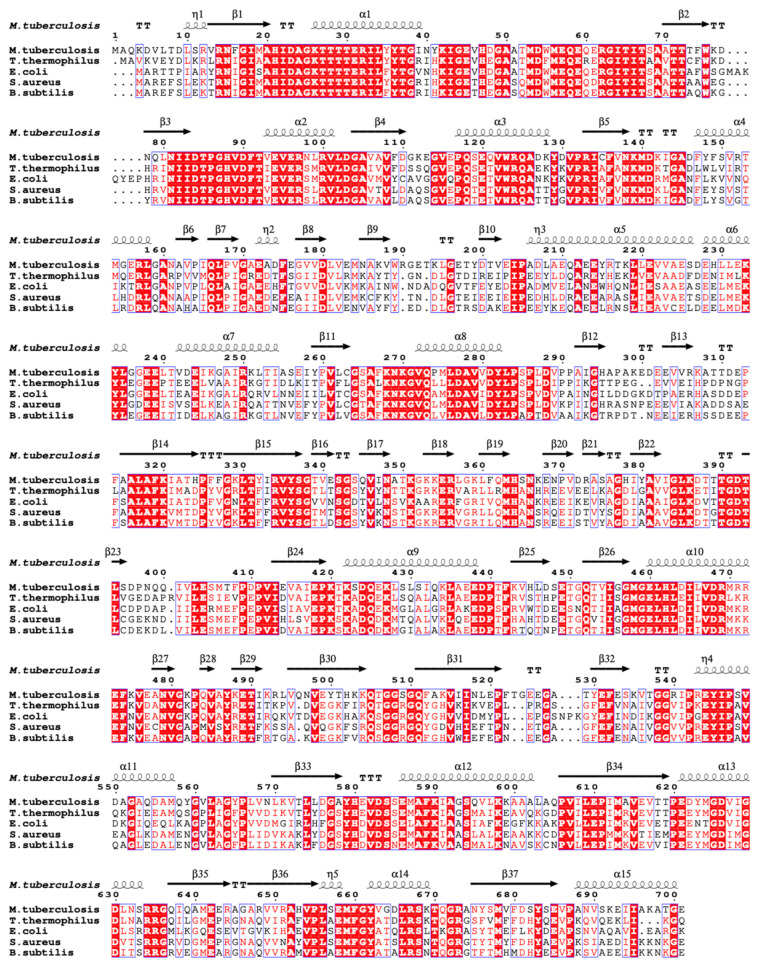
EF-G sequence alignment among different species using ENDscript server [53]. Identical and similar residues among groups are shown in white font on a red background and in red font on a white background, respectively.

EF-G sequence comparison among different species showed that *Mtb* EF-G has high sequence similarity (>70%) with *Aspergillus fumigatus* and *Arthrobacter globulus*, but shows 55-58% similarity with *E. coli* and *T. thermophilus* (Figure 6) [68]. The structures of EF-G protein from *T. thermophilus* [65], *E.coli* [70], Mtb [68], *Staphylococcus aureus* [63], and *Bacillus subtilis* (PDB:5VH6) have been reported. Among them, the complex structures of EF-Tu with different components including ribosome, GDP, GDP analogs, tRNA, and RRF in *T. thermophilus* and *E. coli* were obtained by X-ray crystallography or electron microscopy methods (Table 3).

**Figure 7:**
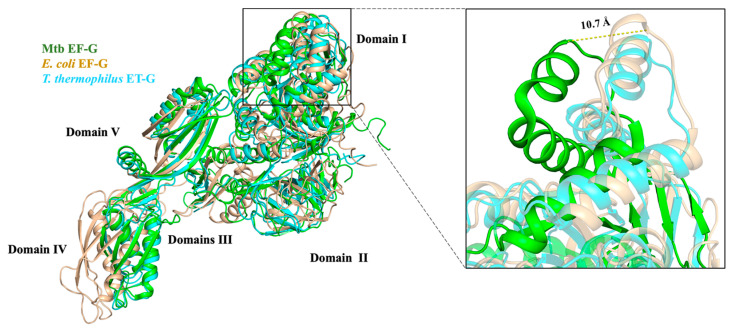
EF-G structure alignment among Mtb (PDB:7CDW, green), *E. coli* (PDB:3J9Z, light orange), and *T. thermophilus* (PDB:1DAR, cyan). The zoomed-in area shows the detailed difference of domain I among three species (residues 160–256). The α5 and α6 helices of Mtb EF-G shifted about 10.7 Å compared to the other two species.

**Figure 8:**
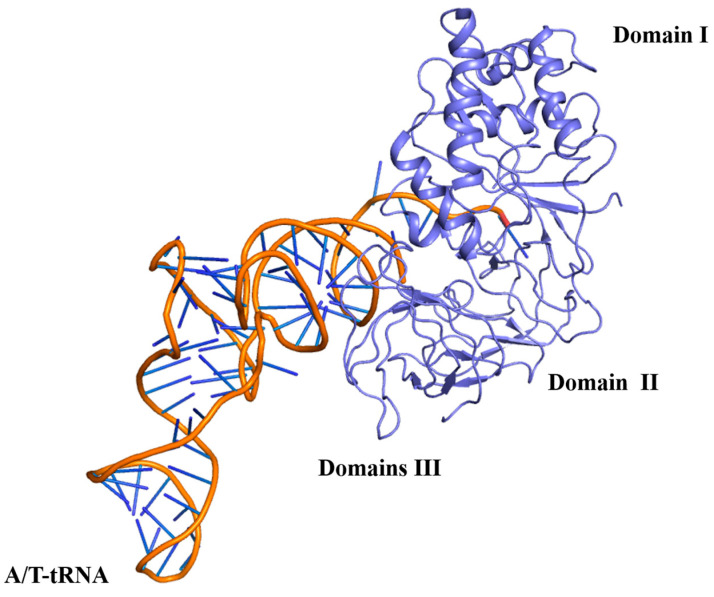
The complex of aminoacyl-tRNA−EF-Tu−GTP(PDB:2WRN) has a highly similar domain organization to EF-G.

The structural superimposition showed that the EF-G proteins among Mtb, *E. coli*, and *T. thermophilus* have a similar architecture. Although the RMSD value between Mtb EF-G and *E. coli* EF-G is 8.120 Å, the RMSD value between Mtb EF-G and *T. thermophilus* EF-G is 4.099 Å. All three EF-G proteins have five structural domains, from the N-terminus to the C-terminus named domain I (also known as the G domain), domain II, domain III, domain IV, and domain V (Figure 7). Domains I, II, III, and V are responsible for GTPase activity and ribosome binding. Domain IV of EF-G acts as a mediator in the translation [10]. The five domains fold into an elongated shape (Figure 7) [68] that is similar to the ternary complex of aa-tRNA-EF-Tu·GTP [10]. This is probably the best known example of molecular mimicry, and emphasizes the need for both the EF-G and the aa-tRNA-EF-Tu·GTP ternary complex to occupy a similar position at the ribosomal subunit interface [10] (Figure 7 and Figure 8). In addition, structural domain I (residues 2-292) of Mtb EF-G is very similar to the structures of other GTPases, except for the insertion of 90 residues (residues 160-256) (Figure 7) [68].

FA and argyrin B are two drugs that bind to different sites of EF-G [71]. FA is a widely used clinical antibiotic against EF-G in Gram-positive bacteria and shows anti-tuberculosis activity in vitro [71,72,73]. Structural modeling of FA with Mtb EF-G showed that the FA binding pocket is surrounded by domains G, II, and III, similar to the binding pocket of *T. thermophilus* [68]. Argyin B, a cyclic octapeptide with antimicrobial activity against Gram-negative pathogens, also targets EF-G through a novel mechanism [74]. During translocation, the cyclic octapeptide antibiotic argyrin B traps EF-G on the ribosome, thereby inhibiting translation [75].

### 2.4. Computer-Aided Structure-Based Anti-Tuberculosis Drug Design

Tuberculosis remains a major global public health problem, and the rapid discovery and development of new anti-tuberculosis drugs is urgent. Traditional drug discovery methods are time-consuming, costly, and labor-intensive. Based on the understanding of the quantitative relationship between structure and biological activity, computer-assisted drug design applications have emerged, which have several advantages such as affordability, targeting, and predictability compared to traditional drug design methods [76,77]. Novel drug design is divided into two categories: (1) receptor/enzyme-based drug design and (2) ligand-based drug design [78]. The former approach is currently more popular. Creating suitable small molecules for enzyme-based design requires high-quality target protein structures and precise knowledge of the protein’s active site [76]. This approach entails designing small molecules by matching fragment molecules to the binding pocket of the target protein. Co-crystallization of ligands and receptors after rational computer analysis of the binding pocket would be more efficient. Often regarded as the most important part of structure-guided drug design, X-ray crystal structures provide important insights for the development of new drugs [76].

The discovery of novel structural classes of antibiotics is urgently needed to address the current antibiotic resistance crisis. Deep learning methods can help discover new antibiotic drugs [79]. Based on computer-aided drug design and interpretable deep learning models, Wong et al. identified a revolutionary new type of antibiotic, among more than 12 million compounds, that can kill methicillin-resistant *Staphylococcus aureus* (MRSA), a common clinical superbug. Using a deep learning-guided method, Liu et al. identified an antibiotic that targets *Acinetobacter baumannii* [80]. Moreover, these compounds have low toxicity to human cells, making them excellent antibiotic candidates [81]. This approach is likely to pay off well in the future if applied to the modification of anti-tuberculosis antibiotics.

The structures of several key proteins of Mtb have been elucidated, e.g., membrane transporter MmpL3 [82,83], RNase J [84], fatty acyl-AMP ligase FadD32 [85], acetyltransferase Eis [86], EF-Tu/EF-Ts [49], EF-G [68], and nucleoside triphosphate pyrophosphohydrolase MazG [87]. Inhibitors of these proteins are considered promising for the treatment of tuberculosis, among which 11 potential anti-tuberculosis drugs have been obtained by Li et al., based on the rational drug of acetyltransferase Eis [86]. Hu et al. designed anti-mycobacterial drugs that target MmpL3 using a structure-based drug design [83]. Translation elongation factors have been considered key drug targets. However, due to the late report on the structure of EF-G [68], EF-Tu, and EF-Ts [49] of Mtb, the study of new antibiotics based on these three structures is yet to emerge, but is already underway. Obtaining the structure of the protein-antibiotic complexes in the future will probably provide numerous aids.

## 3. Conclusions

Tuberculosis is one of the deadliest diseases that affects human society. Translation elongation factors are important drug targets, and several anti-tuberculosis antibiotics targeting translation elongation factors have been reported. The structures of Mtb EF-G, EF-Tu, and EF-Ts could provide theoretical bases for the rational design and development of new molecules with novel/unique modes of action in the future [49,68]. Currently, modifications for antibiotics based on structure are already underway. For example, to prevent FA from being rapidly metabolized, a team of researchers chemically modified FA and obtained an in vivo stable and biologically characterized FA derivative C-3 silicate, which was found to have antimycobacterial activity comparable to that of FA [71]. Moreover, the structure of the Mtb ribosome might provide an exquisite structural basis for the study of Mtb translation [88]. Solving the structure complex of the Mtb ribosome with EF-G and its derivative will further provide valuable insights into the design of FA analog inhibitors against EF-G.

Translation elongation factors not only play essential roles in the normal translation process, but are also important participants in trans-translation. Because of the ubiquity and critical role of trans-translation in bacteria, trans-translation inhibitors have great potential to be effective broad-spectrum antibiotics. Additionally, as trans-translation pathways have not been identified in animals, inhibitors targeting trans-translation are less likely to cause adverse effects in the host. Thus, trans-translation is becoming an important target for antibiotic development [4]. The complex structures of *E. coli* tmRNA-SmpB-EF-G [66], the EF-Tu-tmRNA [16], or the EF-Tu-tRNA [89], as well as the *T. thermophilus* EF-Tu-tmRNA [89] and EF-G-tRNA [90], have been reported recently. Moreover, Marathe et al. showed that antibiotics that inhibit translation in *E. coli* block the binding of EF-Tu to tmRNA, but not the binding of tRNA to EF-Tu [4]. Analyses of the mechanisms of translation elongation factors during normal ribosome translation and trans-translation rescue translation are likely to provide new ideas for drug development.

Although highly desirable, the complex structures of tRNA or tmRNA binding to EF-Tu or EF-G of Mtb have not yet been reported. Addressing these issues will help us develop new anti-tuberculosis drugs in the future. On the other hand, computer-aided structure-based methods might provide endless opportunities to explore drug discovery and design, including, but not limited to, predicting protein binding pocket, molecular docking, and ADMET [76,91]. Many of them have also been applied in the development of anti-tuberculosis drugs, such as drugs targeting MurB and MurE enzymes [92], flavoenzyme DprE1 [93], dTDP-4-dehydrorhamnose reductase (RmlD) [94], LipU protein [95], and enoyl-acyl carrier protein reductase [96]. As more Mtb-derived proteins are structurally analyzed and the resources of drug design platforms are further enriched, computer-aided structure-based drug design may not only alleviate the enormous burden that TB places on the world’s healthcare systems, but may also be extended to develop drugs targeting proteins of other pathogenic bacteria.

## Figures and Tables

**Table 1:** Representative EF-Tu structures from different bacterial sources.

Source	Components	PDB ID	Resolution (Å)	Method
*E. coli*	EF-Tu + L11 + S12 + S13	1MJ1	13.0	EM
	EF-Tu + S12 + L11	3EQ3; 3EQ4	9.0; 12.0	EM
	EF-Tu + Ribosome	1QZA; 1QZB; 1QZD	10.0	EM
	EF-Tu + Ribosome + SmpB	7ABZ	3.2	EM
	EF-Tu + GE2270A	1D8T	2.35	X-ray
	EF-Tu + GDP	1DG1; 1EFC; 1ETU	2.5; 2.05; 2.9	X-ray
	EF-Tu + GDP	1EFM; 2FX3; 2HCJ	2.7; 3.4; 2.12	X-ray
	EF-Tu + GDP + KKL55	8FR3	2.23	X-ray
	EF-Tu + EF-Ts	1EFU; 4PC3; 4PC6	2.5; 1.83; 2.2	X-ray
	EF-Tu + GNP + kirromycin	1OB2	3.35	X-ray
	EF-Tu + GNP + enacyloxin IIa	2BVN	2.3	X-ray
*M. tuberculosis*	EF-Tu + EF-Ts	7VMX	2.8	X-ray
	EF-Tu + GDP	7VOK	3.4	X-ray
*T. thermophilus*	EF-Tu + Ribosome + SmpB	1ZC8	13.0	EM
	EF-Tu + Ribosome + GNP	2P8W	11.3	EM
	EF-Tu + Ribosome + kirromycin	4V68	6.4	EM
	EF-Tu + Ribosome + GCP	4V5L	3.1	X-ray
	EF-Tu + EF-Ts	1AIP	3.0	X-ray
	EF-Tu + GNP	1EXM	1.7	X-ray
	EF-Tu + GNP + GE2270 A	2C77; 2C78	1.6; 1.4	X-ray
	EF-Tu + Aurodox	1HA3	2.0	X-ray
*T. aquaticus*	EF-Tu + GDP	1B23; 1TUI	2.6; 2.7	X-ray
	EF-Tu + GNP	1EFT; 1TTT	2.5; 2.7	X-ray
	EF-Tu + GNP + enacyloxin IIa	1OB5	3.1	X-ray
*P. aeruginosa*	EF-Tu + Tse6 + GDP	4ZV4	3.5	X-ray
*P. putida*	EF-Tu + GDP	4J0Q	2.29	X-ray
*S. solfataricus*	EF-Tu + GDP	1JNY; 1SKQ	1.9; 1.8	X-ray

**Table 2:** Structures of the complexes of antibiotics with EF-Tu.

Source	Proteins	Antibiotics	PDB ID	Binding Domain
*E. coli*	EF-Tu	Kirromycin	1OB2	Domain I & III
	EF-Tu	Enacyloxin IIa	2BVN	Domain I & III
*T. thermophilus*	EF-Tu	GE2270A	2C77	Domain II
	EF-Tu	Pulvomycin	2C78	Domain II

**Table 3:** Representative EF-G structures from different bacterial sources.

Source	Components	PDB ID	Resolution (Å)	Method
*E. coli*	Ribosome + EF-G + GDP	4V7B	6.8	EM
	50S + EF-G + GDP analogs + RRF	2RDO	9.1	EM
	S12 + EF-G + RRF	3J0E	9.9	EM
	Ribosome + EF-G + tRNA	4V7D	7.6	EM
	Ribosome + EF-G + GTP	3J9Z; 3JA1	3.6	EM
	EF-G + RRF	1PN6; 1ZN0	15.5	EM
*T. thermophilus*	EF-G + GDP	1FNM; 1DAR	2.8; 2.4	X-ray
	EF-G + GDP	1EFG; 2EFG	2.7; 2.6	X-ray
	EF-G + GDP	2BM0; 2BM1	2.4; 2.6	X-ray
	EF-G + GNP	2BV3; 2J7K	2.5; 2.9	X-ray
	Ribosome + EF-G + GDP	4V8U; 4V90	3.7; 2.95	X-ray
	EF-G + L11	1JQM;1JQS	18.0	EM
	EF-G + Ribosome + GDP	4V5M; 4V5N	7.8; 7.6	EM
*M. tuberculosis*	EF-G + GDP	7CDW	3.0	X-ray
*S. aureus*	EF-G	2XEX; 3ZZ0; 3ZZT;	1.9; 2.8	X-ray
*S. aureus*	EF-G	3ZZU	2.95; 2.9	X-ray
*B. subtilis*	EF-G	5VH6	2.61	X-ray
*E. faecalis*	EF-G	6BK7	1.83	X-ray
